# Antimicrobial activities of graphene oxide against biofilm and intracellular *Staphylococcus aureus* isolated from bovine mastitis

**DOI:** 10.1186/s12917-022-03560-6

**Published:** 2023-01-14

**Authors:** Shamsaldeen Ibrahim Saeed, Liang Vivian, C. W. Salma C. W. Zalati, Nani Izreen Mohd Sani, Erkihun Aklilu, Maizan Mohamad, An’ Amt Mohamed Noor, Kasturi Muthoosamy, Nor Fadhilah Kamaruzzaman

**Affiliations:** 1 Nanotechnology in Veterinary Medicine (NanoVet) Research Group, Faculty of Veterinary Medicine, University Malaysia Kelantan, Kelantan 16100 Pengkalan Chepa, Malaysia; 2 Faculty of Veterinary Science, University of Nyala, PO Box 155, Nyala, South Darfur State Sudan; 3 Faculty of Bioengineering and Technology, Universiti Malaysia Kelantan, 17700 Jeli, Malaysia; 4 Nanotechnology Research Group, Centre of Nanotechnology and Advanced Materials, University of Nottingham Malaysia, 43500 Semenyih, Selangor Malaysia

**Keywords:** Graphene oxide, Mastitis, Intracellular *S. aureus*

## Abstract

**Background:**

*S. aureus* is one of the causative agents of bovine mastitis. The treatment using conventional antimicrobials has been hampered due to the development of antimicrobial resistance and the ability of the bacteria to form biofilms and localize inside the host cells.

**Objectives:**

Here, the efficacy of graphene oxide (GO), a carbon-based nanomaterial, was tested against the biofilms and intracellular *S. aureus invitro*. Following that, the mechanism for the intracellular antimicrobial activities and GO toxicities was elucidated.

**Methods:**

GO antibiofilm properties were evaluated based on the disruption of biofilm structure, and the intracellular antimicrobial activities were determined by the survival of *S. aureus* in infected bovine mammary cells following GO exposure. The mechanism for GO intracellular antimicrobial activities was investigated using endocytosis inhibitors. GO toxicity towards the host cells was assessed using a resazurin assay.

**Results:**

At 100 ug/mL, GO reduced between 30 and 70% of *S. aureus* biofilm mass*,* suggesting GO’s ability to disrupt the biofilm structure. At 200 ug/mL, GO killed almost 80% of intracellular *S. aureus,* and the antimicrobial activities were inhibited when cells were pre-treated with cytochalasin D, suggesting GO intracellular antimicrobial activities were dependent on the actin-polymerization of the cell membrane. At < 250 ug/mL, GO enhanced the viability of the Mac-T cell, and cells were only affected at higher dosages.

**Conclusion:**

The in vitro efficacy of GO against *S. aureus* in vitro suggested the compound could be further tested *in Vivo* to zrecognize its potential as one of the components of bovine mastitis therapy.

## Introduction

Bovine mastitis is the inflammation of the mammary gland. It is considered the most frequent disease in dairy cattle, leading to substantial economic loss in dairy industries due to reduced milk production, increased death and culling rates, and increased treatment costs [1, 2]. Mastitis is estimated to cost the global dairy industry US$19.7 to US$32 billion annually [3]. The United States estimates US$2 billion in losses to mastitis in the dairy industry annually [4]. Mastitis is the multi-etiological disease that can be caused by various microorganisms such as bacteria, fungi, viruses, and algae [5]. It is estimated that more than 150 bacterial species can cause mastitis in dairy cattle which include the bacteria *S. aureus*, *Mycoplasma* spp., *Streptococcus uberis, Streptococcus dysgalactiae, Escherichia coli and Klebsiella pneumonie* [6]. Among them, *S. aureus* has been correlated with at least 50% of subclinical mastitis cases [6]. *S. aureus* is part of the normal microbiota of the udder skin and teat and can enter the teat canal and colonize the internal components of the mammary gland. Intramammary infection by *S. aureus* is the primary cause of subclinical mastitis, resulting in a persistent chronic infection [5].

Antimicrobials are widely used to treat and control bovine mastitis [7, 8]. The common antimicrobials used in dairy farms include beta-lactams, extended beta-lactams, macrolides, tetracycline, fluoroquinolones, and sulfonamides, which were administered mainly via intramammary and intramuscular routes [9]. However, the treatment has only been partially effective, as the cure percentage only ranges from 10 to 30% [7]. The poor outcome of the existing treatment is, in part, due to the emergence of *S. aureus* antimicrobial resistance (AMR), which reduces antimicrobial efficacy. AMR *S. aureus* has been isolated from bovine milks worldwide, where 60 to 90% of *S. aureus* isolated from clinical mastitis and milk sample were resistant to β-lactam antimicrobials [10–16]. Not only causing mastitis and economic problem but*but S. aureus has also* been recognized as a serious public health risk, considering the emergence of the livestock-associated MRSA (LA-MRSA) clonal complex 398 strain that has been associated with human infections [17].

Also, *S. aureus* is known to produce biofilms, a cluster of bacterial communities within the extracellular matrix that consists of glycolipids, deoxyribonucleic acid (DNA), proteins, lipopolysaccharides, and other bacterial secretion compound [16, 18]. This structure provided physical barriers to the bacteria that decreased the antimicrobial penetration and efficacy. Besides, the extracellular DNA and polysaccharides can interact with antimicrobials and further prevent their penetration across the structure [19, 20]. Additionally, *S. aureus* is known to invade and survive within the host cells [16]. The intracellular survival of *S. aureus* in mammary epithelial cells has been associated with subclinical bovine mastitis and reinfection in treated dairy cows [21]. Therefore, AMR, the presence of *S. aureus* as biofilms, and its ability to invade the host cells provide additional privileged reservoirs for the bacteria as it is protected from the host immune responses and the effect of antimicrobials [22]. This, in turn, causes re-infection, resulting in the long-term disease course that continues to pose a huge treatment challenge for the dairy industries.

**Fig. 1:**
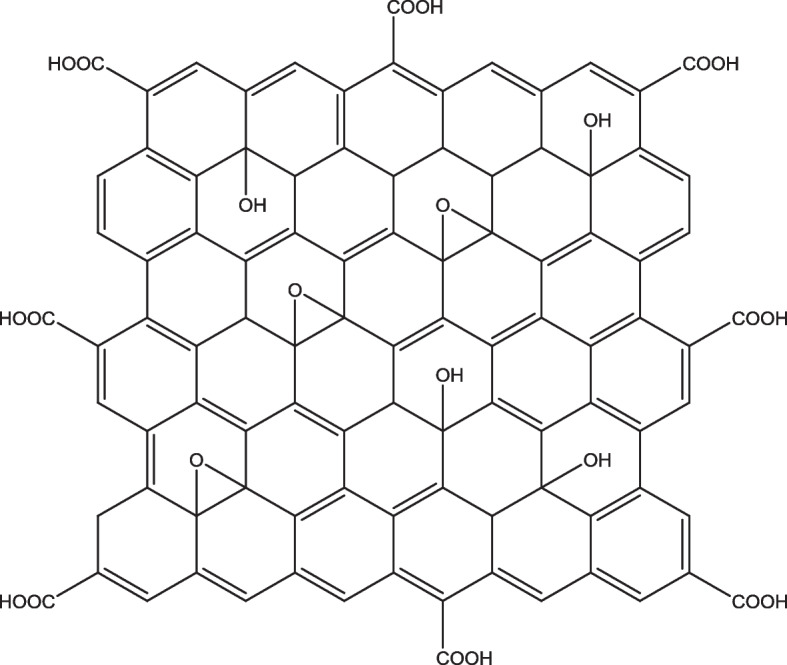
The structure of gaphene oxide (GO). GO consists of both sp^2^ carbons and oxygen-containing functional groups, such as hydroxyl (−OH), epoxy (C-O-C), carbonyl (C=O), and carboxylic acid (−COOH) [28]

Therefore, there is an increasing need to evaluate and develop alternative methods for the effective treatment of bovine mastitis. A wide range of alternatives been developed by researchers for the treatment of bovine mastitis. These include the application of antimicrobial peptides, bacteriophage therapy, nanomaterials, and natural products from plants and animals [23–26]. Recently, the development of carbon-based nanomaterial such as graphene has received great attention to be used as an antimicrobial agent in medical applications due to its considerably less toxicity, ease of production and functionalization as well as high solubility in aqueous media [27]. Graphene derivatives such as graphene oxide (GO) pose innate antimicrobial activities due to their unique physicochemical properties. GO is the oxidized form of graphene, consisting of sp^2^ carbons and an oxygen-containing functional group (Fig. 1) [29]. The presence of oxygen functional groups such as carbonyl (C=O), the carboxylic acid (−COOH), hydroxyl (−OH), alkoxy (C-O-C) allows interaction of the compound with bacteria and other biomolecules, making it an interesting candidate for antimicrobial therapy. The present study aims to investigate the antimicrobial activities of graphene oxide towards biofilm and intracellular *S. aureus.* Following that, the mechanism of the intracellular antimicrobial activity and the cytotoxicity of the compound towards bovine mammary epithelial cells were determined.

## Materials and methods

### Graphene oxide preparation and characterization

Graphene oxide (GO) was purchased from (GO Advanced Solutions Sdn. Bhd. Malaysia). For stock preparation, GO was dried, weighed, and resuspended in water. Prior to experiments, GO was re-diluted in water according to the designated concentration. GO was analyzed using the FFourier transform infrared spectroscopy (FTIR, Themo, USA) to investigate the surface functional groups on GO.

### Bacterial isolates and growth condition

Three isolates of *S. aureus* obtained from the subclinical bovine mastitis in east coast Malaysia were included in this study. These isolates were chosen from a pool of clinical isolates that were screened based on their ability to invade the host cells. The isolates were kept in 20% glycerol stock at − 80 °C. When required, the bacteria stock was re-cultivated in trypticase soy broth (Difco Laboratories, Detroit, MI) for 18 h at 37 °C. The overnight cultures were then transferred to Mannitol salt agar (MSA) and cultured for 18 h at 37 °C. Single colonies were isolated and regrown in broth and proceeded according to the subsequent experiment.

### Antimicrobial activity of GO

The antimicrobial activity of GO towards extracellular *S. aureus* was evaluated by using time-kill methods [30]. Briefly, the diluted bacterial cells were incubated with different concentrations of GO (50, 100, 200, 400, and 600 μg/mL) at 37 °C in an incubator shaker (200 rpm) for 2 and 4 h. Following that, 100 μL aliquots were taken out, serially diluted in saline, plated on nutrient agar plates, and incubated for 18 hours at 37 °C, followed by colony counting to estimate the percentage of bacteria viability correspondence to the untreated bacteria. All experiments were performed in triplicate. The data were expressed in CFU/mL.

### Antibiofilm activities of GO

Biofilm formation assays were conducted on *S. aureus* isolates according to the protocol described in our previous work [31]. Three biofilm-producing *S. aureus* were selected for testing GO antibiofilm activity. Biofilms were exposed to different concentrations of GO for 3 h, followed by staining with crystal violet solution and measurement of the biofilms by spectrophotometer. Then the percentage of remaining biofilm was calculated and compared with the untreated biofilm, corresponding to 100% biofilm mass.

### Mammalian cells culture and growth conditions

Bovine mammary epithelial cell (Mac-T cells) was used as the model of bovine udder cell and host for the intracellular infection. Cells were obtained from Prof Liam Good, Royal Veterinary College, London. Cells were maintained according to the protocol described in our previous work [31].

### Graphene oxide (GO) intracellular killing activities

Intracellular infections of Mac-T cells (host cells) by *S. aureus* were established using a gentamicin protection procedure described by Kamaruzzaman et al. [31–34]. Briefly, Mac-T cells were infected by the bacteria for 3 h, followed by the treatment of cells with gentamicin to kill extracellular bacteria. Following that, a range concentration of GO was added for another 3 h. GO was removed, and cells were rinsed and lysed. Lysed cells were serially diluted and plated on nutrient agar. Viable bacteria were enumerated as CFU/mL. For each experiment, gentamicin-treated cells (without GO treatments) were used as controls.

### Elucidation the mechanism of GO intracellular antimicrobial activities

Gentamicin protection assays were performed as mentioned above. Prior to treatment with GO, *S. aureus* infected Mac-T cells pre-treated with different endocytosis inhibitors; dynasor hydrate (26 μg/mL), cytochalasin D (5 μg/mL), and ikarugamycin (5 μg/mL) for 15 min. Then, the medium containing the inhibitor was removed, and cells were washed with PBS and proceeded with treatment with GO (100 μg/mL) for another 3 h. Following that, GO solution was removed, and cells were rinsed and lysed. Lysed cells were serially diluted and plated on nutrient agar. For each experiment, GO treated cells (without endocytosis inhibitor treatments) were used as controls.

### Cytotoxicity assay

Resazurin assay was used to assess the toxicity of GO towards Mac-T cells according to the protocol described in our previous work [31]. In brief MAC-T cells (4 × 10^4^ cells/well) were added to a 96 well plate and incubated at 37 °C for 24 hours in the presence of CO_2_. After 24 hours, cells were incubated with 100 μl of GO solutions at different concentrations at 37 °C for 3 hours. Non-treated cells and medium only were used as controls. Resazurin sodium salt (Sigma-Aldrich, UK) was prepared as a stock solution at 440 μM in PBS and added to each well at 44 μM final concentration, and plates were incubated for an additional 48 hours. Optical density (OD) was measured using POLARstar Omega microplate reader (BMG, Labtech, Germany) at 550 nm and 630 nm. The OD value change (or % dye reduction) is proportional to the viable cell number and was used to calculate the concentration of GO that can inhibit 50% of MAC-T cells or called half-maximal inhibitory concentrations (IC50). All experiments were conducted in triplicate.

### Statistical analysis

Statistical analysis was performed using GraphPad Prism 8 (San Diego, CA, USA). A one-way analysis of variance (ANOVA) was choosing for analysis. Data were presented as means ± standard deviation (SD). The level of significance was accepted as *p* ≤ 0.05. For the graph, error bars represent standard deviations. *p ≤ 0.05; *** *p* ≤ 0.001; **** *p* ≤ 0.000; and NS, not significant. All experiment was performed at least three times.

## Result

### GO antimicrobial activities against extracellular *S. aureus*

**Fig. 2:**
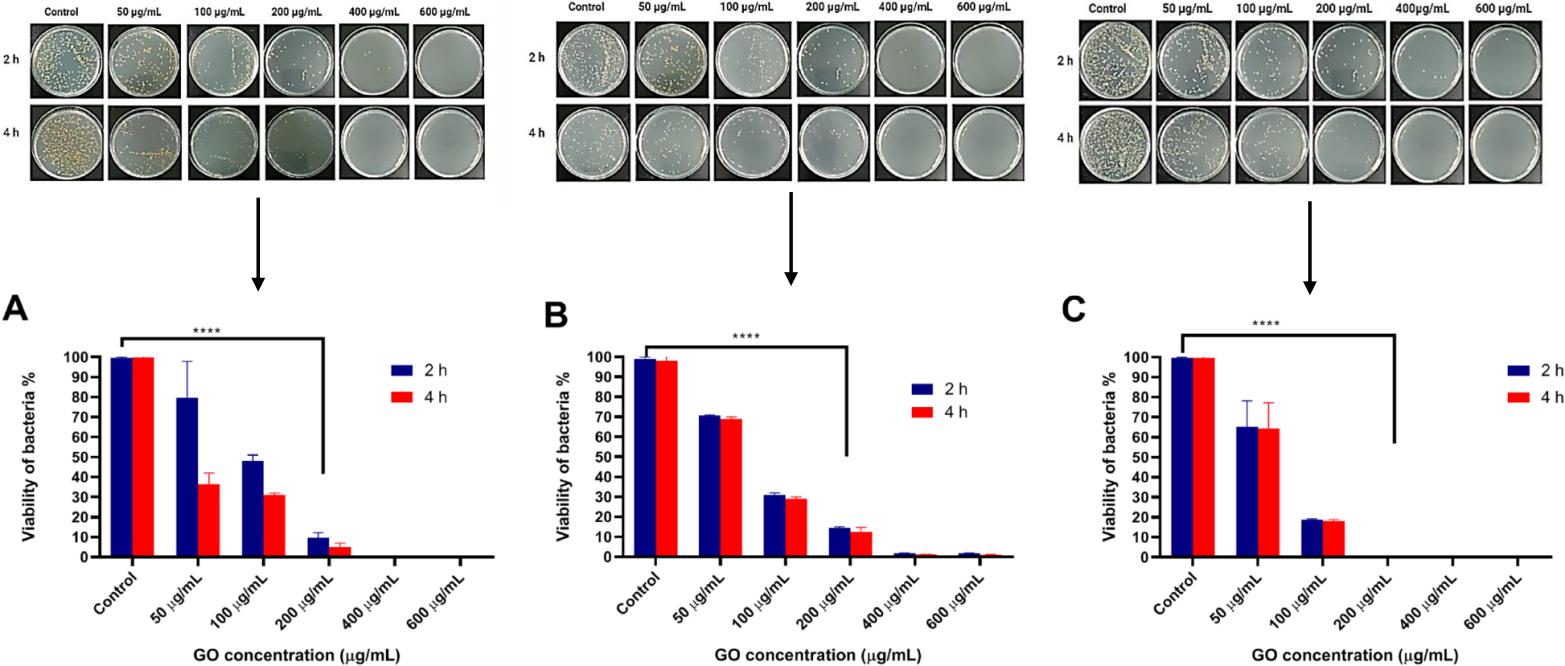
Antimicrobial activity of GO against extracellular *S. aureus* F31D (**a**); *S. aureus* F41B (**b**) and *S. aureus* F53D (**c**) at different concentrations and time of exposure. Error bars represent standard deviation of triplicates. *p ≤ 0.05; *** *p* ≤ 0.001; **** *p* ≤ 0.000; and NS, not significant

GO at a 200 μg/mL reduced 90% of bacterial cell viability for all tested isolates. GO antimicrobial action was highly dependent on the concentration and exposure time, as the viability of bacteria cells decreased with increased GO concentration and incubation time. Figure 2 summarises the bacteria viability after being treated with GO for 2 and 4 h, respectively.

### *S. aureus* intracellular infection model

**Fig. 3:**
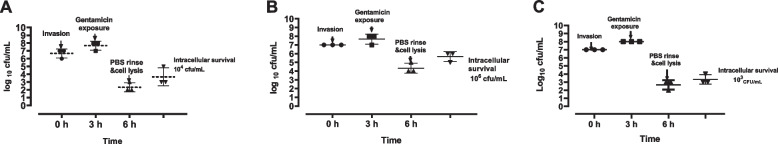
Gentamicin protection assay of *S. aureus* in Mac-T cells. Following gentamicin exposure, lysis of Mac-T cells released approximately **a**) 10^4^ CFU/mL of *S. aureus* F31D, **b**) 10^6^ CFU/mL of F41B, and **c**) 10^3^CFU/mL of F53D. Error bars represent standard deviation of triplicates. *p ≤ 0.05; *** *p* ≤ 0.001; **** *p* ≤ 0.000; and NS, not significant

To determine GO antimicrobial activity against intracellular *S. aureus*, a gentamicin protection assay was performed for the three *S. aureus* isolates using Mac-T as the host cells. These isolates were confirmed to invade Mac-T cells, as indicated by the increasing survival of *S. aureus* following gentamicin exposure and cell lysis. Lysis of Mac-T cells released approximately 10^4^ CFU/mL of *S. aureus* F31D, 10^6^ CFU/mL of F41B, and 10^3^ CFU/mL of F53D that were protected from gentamicin antibacterial activities as depicted in Fig. 3.

### GO antibiofilm activities

**Fig. 4:**
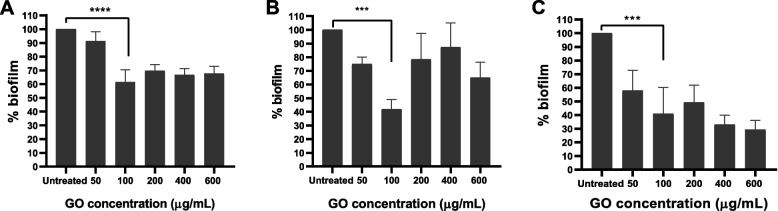
GO activity against biofilms of *S. aureus* **a)** F03B **b)** F41B, and **c)** F53D. Error bars represent standard deviation of triplicates. *p ≤ 0.05; *** *p* ≤ 0.001; **** *p* ≤ 0.000; and NS, not significant

GO demonstrated antibiofilm activities, where a reduction of biofilm mass was observed when exposed to the compound. GO at 100 μg/mL reduced 30–70% of biofilms’ mass following GO exposure, as demonstrated in Fig. 4.

### Antimicrobial activities of GO against intracellular *S. aureus*

**Fig. 5:**
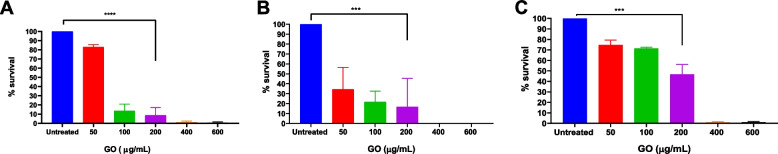
Antimicrobial activity of GO against intracellular *S. aureus* strains **a**) F31D, **b**) F41B, and **c**) F53D. Untreated infected cells were used as a control to establish CFU value corresponding to 100% survival. Error bars represent standard deviation of triplicates. *p ≤ 0.05; *** *p* ≤ 0.001; **** *p* ≤ 0.000; and NS, not significant

To determine the GO antimicrobial activities against intracellular *S. aureus*, infected host cells were treated with different concentrations of GO prepared in 0.9% NaCl (50, 100, 200, 400, and 600 μg/mL) for 3 h. GO was found effective in killing intracellular bacteria, as evidenced by the reduction of viable bacteria following the treatment. GO at 200 μg/mL killed 20–80% of intracellular *S. aureus*. However, GO efficacies varied between each tested isolate. Nonetheless, at higher concentrations, > 200 μg/mL, GO was able to kill between 50 to 100% intracellular bacteria for all strains tested. Figure 5 shows the percentage of survival of *S. aureus* after treatment with different concentrations of GO compared to the untreated infected cells.

### The influence of solvent on GO intracellular antimicrobial activities

**Fig. 6:**
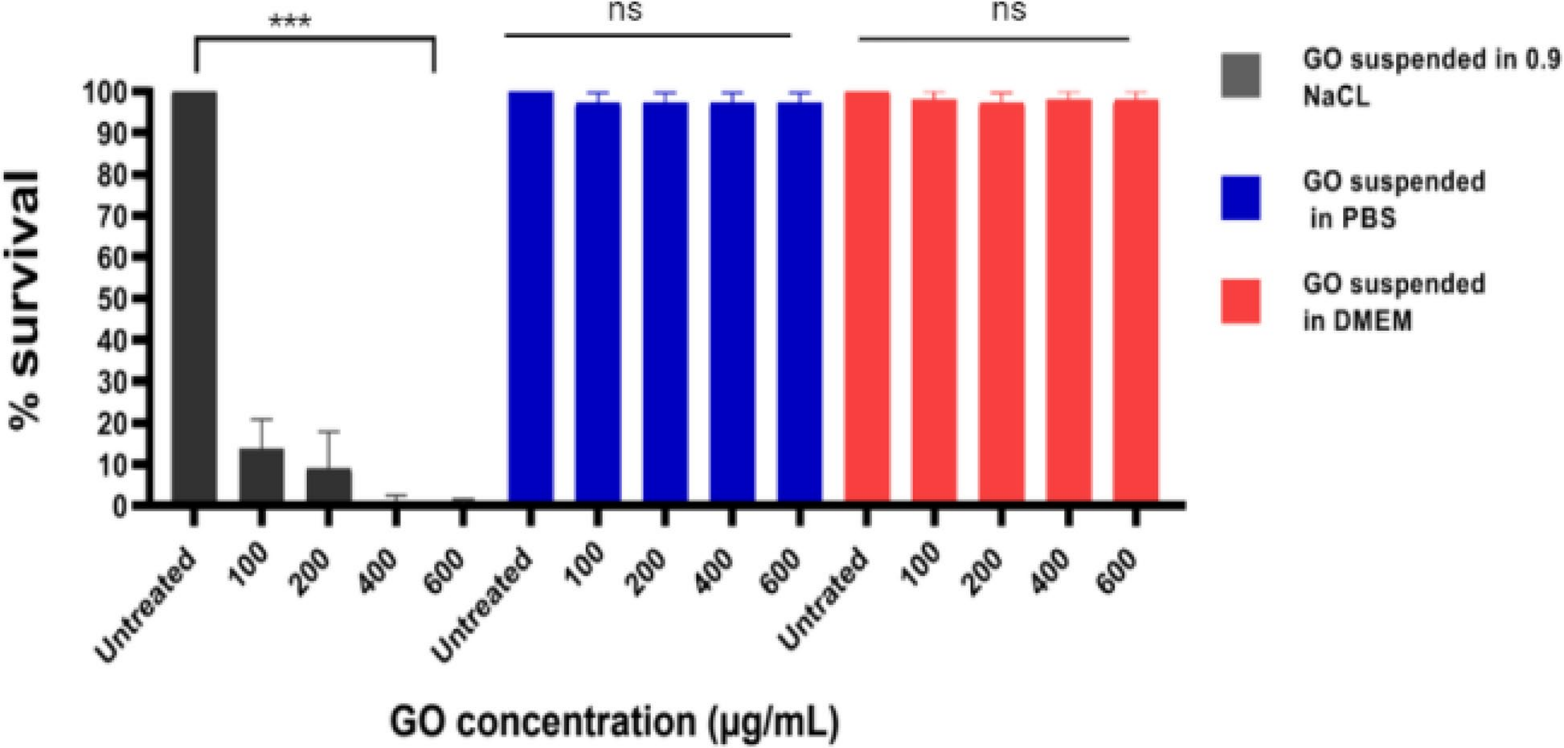
The influence of 0.9% NaCl, PBS*,* and DMEM towards GO intracellular antimicrobial efficacy. Error bars represent *the* standard deviation of triplicates

To determine if GO intracellular antimicrobial activities could be influenced by types of solvents used during the experiment, infected host cells were treated with various concentrations of GO suspended in three different solvents; 0.9% NaCl, phosphate buffer saline (PBS) and dulbenco modified eagles medium (DMEM). The infected cells were exposed to the GO solution for 3 h in the designated solvent. GO suspended in NaCl demonstrated efficient intracellular killing activities compared to the GO suspended in PBS and DMEM, highlighting the influence of the solvent on GO antimicrobial activities Fig. 6.

### Mechanism of GO intracellular antimicrobial activities in Mac-T cells

To investigate the possible mechanism for GO intracellular antimicrobial activities inside Mac-T cells, infected cells were pre-treated with different endocytosis inhibitors; dynasor hydrate, cytochalasin D and ikarugamycin before being exposed to GO. Dynasor hydrate is the inhibitor for the dynamin uptake process, cytochalasin D is the inhibitor for the actin polymerisation of the membrane, and ikaguramycin is the clathrin dependent inhibitor; all are the specific endocytic route for macromolecule uptake into the mammalian cells. In order for GO to be effective against intracellular bacteria, the compound needs to be taken up by the cells, mainly via endocytosis route. If GO intracellular antimicrobials efficacies were reduced by a specific endocytosis inhibitor, that could suggest the inhibitor blocked the specific uptake route for the compound into the bovine mammary cells. From the experiment, cells that were pre-treated with cytochalasin D showed reduction in GO intracellular antimicrobial activities compared to cells treated with dynasore and ikaguramycin. Cytochalasin D is a compound that inhibits the micropinocytosis in mammalian cells by actin cytoskeleton depolymerisation of the membrane. Reduction in antimicrobial activities when cell were pre-treated with cytochalasin suggest that GO intracellular antimicrobial activities were dependent on the actin polymerization of the cell membrane that might be responsible for GO uptakes via the micropinocytosis route.

**Fig. 7:**
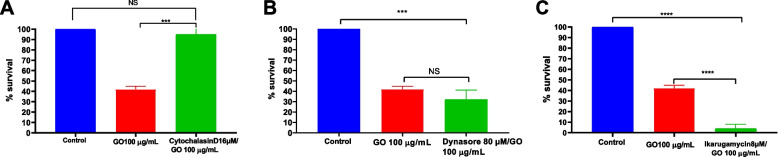
Effect of endocytosis inhibitor towards GO intracellular antimicrobial activities. Infected cells were pre-treated with **a**) cytochalasin D, **b**) dynasore, and **c**) ikarugamycin before exposure to GO (100 μg/mL). Cell pretreated with cytochalasin D reduced GO efficacy against intracellular bacteria, evidenced by increased intracellular bacterial survival. Untreated infected cells (without inhibitors but treated with GO) were used as control to establish the CFU values corresponding to 100% survival. Error bars represent the standard deviation of triplicates. NS (non-significant)

However, it is surprising to observe that cells pre-treated with ikaguramycin showed enhanced GO antimicrobial activities compared to the non-treated cells (Fig. 7). To further confirmed if ikaguramycin alone exhibit antimicrobial activities against intracellular *S. aureus*, the same experiments were repeated, without GO. Indeed, ikaguramycin demonstrated antimicrobial activities against intracellular *S. aureus,* with the effective killing of almost 80% of intracellular *S. aureus* at 5 μg/mL [35].

### Viability of Mac-T cells toward GO

**Fig. 8:**
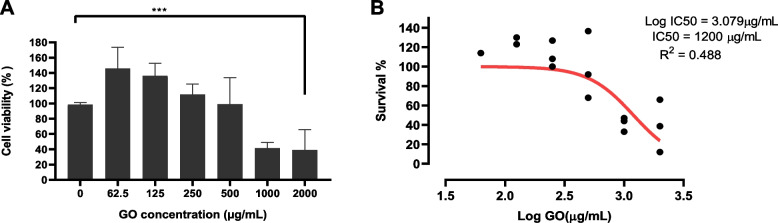
**a **The impact of GO towards Mac-T cells viability; cells viability was affected at concentration > 1000 μg/mL and **b**) Dose-response curve of GO toward Mac-T cells. Error bars represent the standard deviation of triplicates.*p ≤ 0.05; *** p ≤ 0.001; **** p ≤ 0.000; and NS, not significant

Cells exposed to GO between 62.5–250 μg/mL showed enhancement in cell viability compared to the untreated cells. However, cells treated with > 1000 μg/mL showed significant reduction in cell viability. Therefore, this study showed that Mac-T cell viability is highly dependent on the GO concentration. At lower concentrations, GO promotes cell growth, and at higher concentrations, GO causes toxicity toward cell growth. The IC_50_ was determined as 1200 μg/mL; which was higher than the GO concentration required to kill 90% of intracellular *S. aureus* in Mac-T cells (Fig. 8).

## Discussion and conclusion

Here the study demonstrated graphene oxide antimicrobial efficacy against biofilm and intracellular *S. aureus* isolated from subclinical cases mastitis cases in Malaysia. GO was effective against the extracellular, intracellular, and biofilms forms of *S. aureus*. The intracellular antimicrobial activities appeared to be dependent on the actin polymerization of the membrane cell that controls the macropinocytosis uptake pathway of the cells. Finally, the study showed that growth of Mac-T cells was enhanced when exposed to low concentrations of GO, and toxicity was only observed when the cell was exposedto very high concentrationsn of GO.

GO antimicrobial activities could be attributed to several mechanism [26]. GO consist high amount of oxygen functional groups that can wrap the bacteria and, at the same restricting nutrient intake inside the cells [36]. The Van der Walls interaction between the functional groups and the membrane can also trigger oxidative stress, destabilize the bacterial membrane, leading to the release of intracellular contents [37]. In addition, the sharp edges of GO can pierce and thus causing injuries to the membrane structure [38].

GO antibiofilm activities were observed, where reduction of biofilm mass was recorded when GO was exposed to biofilms. This data is consistent with the study conducted by Guilio et al. on the GO effect towards biofilms of *S. aureus, Pseudomonas aeruginosa,* and *Candida albicans*, the most common organisms associated with chronic wounds [39]. Our current study found that GO at 100 μg/mL has better biofilm activity. Whereas at higher concentrations has lower, this could be due to aggregation of GO that could affect their activity. Physiologically, biofilm structure is built of individual cells that are glued together with extra polymeric substances (EPS), which consist of self-released glycolipids, proteins, lipopolysaccharides, and extracellular DNA. The anti-biofilm effect demonstrated by GO could be attributed to the interaction between the hydrogen, Van der Walls, electrostatic, and pi-pi/ π-π interactions. These characteristics allow for interaction with other molecules, including DNA, proteins, and polymers, and destabilize the EPS structure. Also, the sharp edges of GO sheets could penetrate and tear the EPS structure and thus destabilize the microbial biofilm even further.

The treatment for infections by intracellular *S. aureus* remains a challenge because antimicrobials need to penetrate the host cells in order to reach the bacteria. *S. aureus* was reported to zlocalize inside the cell cytosol following the invasion of the host cell [40]. Thus, antimicrobials must not only be able to cross the cell barriers, but it also needs to reach the cytosol where the bacteria reside. Moreover, the mammalian cell membrane is built of the lipid bilayer that allows for selective permeability across its structure [41]. Depending on the physiochemical properties, a compound can either transverse the membrane either through several uptake mechanism. Nanoparticles such as graphene derivatives was reported to be taken up by mammalian cells by several route, depending on the size of the compounds, surface charge as well as the type of cells [41, 42]. For example, graphene quantum dots were reported to be taken up by the breast cancer cells via caveolae mediated endocytosis route [43]. Chen et al. demonstrated that the mechanism for GO uptake into epithelial lung cells, human epithelial skin cell line, human embryonic kidney cell line, and mouse fibroblast cells were size dependent. GO within the size range of 477 nm was taken up by the cells via micropinocytosis, while the smaller sized of GO within 123 nm were mainly taken up by the cells by clathrin and caveolae-mediated endocytosis [44].

In the current study, the uptake mechanism of GO in the bovine mammary cells were indirectly measured by blocking the possible uptake pathway with inhibitors prior to GO exposure toward the infected cell. We anticipated that if GO intracellular antimicrobial were inhibited when cells were pretreated with any of the tested inhibitors, that would suggest the potential route of uptake for the compound into the bovine mammary cells. In this study, reduction in GO efficacy to kill intracellular *S. aureus* were observed when Mac-T cells were pre-treated by cytochalasin D, a macropinocytosis inhibitor, suggesting that the GO uptake into the bovine mammary Mac-T cells were through macropinocytosis route. Macropinocytosis is a clathrin-independent endocytic uptake mechanism for the non-selective solute, including molecules, nutrients, and antigens, that takes place in both phagocytic and non-phagocytic cells [45]. The process involves actin cytoskeleton rearrangement on the plasma membrane triggered by molecules such as GO. The rearrangement can form membrane ruffles that fold back onto themselves, hence trapping the molecule to be transported inside the cells. The invaginated membrane ruffles containing the molecules, e.g., GO then pinched inside the cells within the vesicular membrane. The pinched-off vesicle can become acidified, and this process can trigger the release of GO into the cytosol [45, 46].

Alternatively, there is a possibility for the vesicular membrane to be pierced by GO’s sharpness structure, promoting its release into the cytosol. Another study, however, suggested a different fate for GO. Lammel et al. demonstrated that once GO was taken up by the hepatocellular carcinoma (HEP G2) cells, it was released directly into the cytosol without being trapped in the vesicular compartment [47]. The same observation was also reported when GO was exposed to fish cell lines [48]. Nonetheless, our data on GO’s efficacy against intracellular *S. aureus* suggest that the nanomaterial is able to reach the bacteria inside the host cells to exert antimicrobial activities.

In addition, the GO intracellular antimicrobial activities were highly influenced by the types of solvent or solution used during the experiment, where antimicrobial efficacy was only achieved in saline solution but not in DMEM and PBS. The same observation was reported by Hui et al. that showed GO bactericidal activities against *E. coli* and *Bacillus subtilis* were only recorded in saline but not in Luria Bertani broth, the common medium used for bacteria culture [49]. Another study by Chen et al. showed GO prepared in PBS showed less antimicrobial efficacy compared to GO prepared in NaCl and water when it was tested against *Xanthomonas oryzae pv*. Oryzae [50]. Hui et al. suggested that these phenomena could be due to the absorption of molecules in the medium onto the GO surface, which could have masked its edges and functional groups are responsible for the antimicrobial activities.

Additionally, divalent cations such as Ca^2+^ and Mg^2+^, which is commonly found in the cell culture medium, could cause aggregation in GO molecules, potentially due to cross bridging between the cations and the functional group and edges of the GO sheet [51]. The aggregation could reduce the surface area for the interaction of GO and the bacteria, limiting its antimicrobial efficacy [52]. This important observation, therefore, highlighted the importance of choosing the right solution for the measurement of GO antimicrobial activities.

In this study, GO unexpectedly enhanced bovine mammary cells at low concentrations, and this phenomenon highlighted the versatility of the nanomaterial. The impact of graphene nanocomposites, particularly GO, on cell growth has been recorded before. GO promoted cell growth and induced differentiation of the different types of stem cells, including bone progenitor cells and mouse embryogenic stem cells. Ruiz et al. demonstrated macrophages grown on the surface of low concentration of GO displayed high attachment and promoted cell growth [53]. The increased surface area with functional groups allows for biomolecules and stem cell attachment on the surface. GO has also been shown to support cell growth by allowing spontaneous differentiation and promoting selective differentiation of progenitor cells [54]. Therefore, this observation showed that GO not only effective to kill intracellular *S. aureus*, but it also has the potential to aid bovine mammary cell growth.

This study also demonstrated that bovine mammary cells tolerated GO at concentration that exceeded the bactericidal concentration against the bacteria. This data showed that GO is effective as an antimicrobial at high therapeutic index. Several reports were recorded on graphene toxicity different types of mammalian cells. Nguyen et al. exposed a range of GO concentrations (10 to 200 μg/mL) toward human carcinoma epithelial cell lines. At 200 μg/mL, no obvious toxicity of GO were reported [55]. Several other studies measured GO toxicity towards the following cell line, human breast cancer, ovarian cancer, HeLa and mouse embryonic fibroblast. Briefly, GO toxicity level varied and highly dependent on time of exposure and dose of the compound [55–60]. Nevertheless, this study showed that Mac-T cells appeared to have tolerance to GO with cell viability were only affected when cells were exposed to GO at concentration higher than 1000 μg/mL, which is higher than the concentration needed to kill intracellular *S. aureus* in the host cells.

In conclusion, this study demonstrates that GO has antimicrobial activity against biofilm and intracellular *S. aureus*. GO intracellular antimicrobial activities appeared to be dependent on the actin polymerisation of the Mac-T cells that promotes uptake of the nanomaterial through macropinocytosis route. Finally, GO at low concentration promoted bovine mammary cell growth, and toxicity was only profound when cells were exposed at high concentrations. These findings highlighted GO efficacy and suggested the suitability of the nanomaterial to be further tested and developed as an effective therapy for bovine mastitis.

## Data Availability

All obtained data from this study were included in this manuscript and are available on request from the corresponding author [Shamsaldeen I Saeed].

## References

[1] Wang J (2018). Oligopeptide targeting sortase a as potential anti-infective therapy for *Staphylococcus aureus*. Front Microbiol.

[2] Zhou K, Li C, Chen D, Pan Y, Tao Y, Qu WS (2018). A review on nanosystems as an effective approach against infections of Staphylococcus aureus. Int J Nanomedicine..

[3] Eckersall D, Zadoks R (2016). “Potential biomarkers of mastitis in dairy cattle milk identified” no. July.

[4] Cha E (2011). The cost and management of different types of clinical mastitis in dairy cows estimated by dynamic programming. J Dairy Sci.

[5] Moreira MAS, Júnior AS, Lima MC, Da Costa SL. Infectious diseases in dairy cattle. Elsevier Inc; 2018.

[6] El-Sayed A, Kamel M. Bovine mastitis prevention and control in the post-antibiotic era. Trop Anim Health Prod. 2021;53(2). 10.1007/s11250-021-02680-9. 10.1007/s11250-021-02680-9 33788033

[7] Gomes F, Henriques M (2016). Control of bovine mastitis: old and recent therapeutic approaches. Curr Microbiol.

[8] Cheng WN, Han SG (2020). Bovine mastitis: risk factors, therapeutic strategies, and alternative treatments. Asian-Australasian J Anim Sci.

[9] Abdi RD, Gillespie BE, Ivey S, Pighetti GM, Almeida RA, Dego OK (2021). Antimicrobial resistance of major bacterial pathogens from dairy cows with high somatic cell count and clinical mastitis.

[10] Molineri AI, et al. Antimicrobial resistance of *Staphylococcus aureus* isolated from bovine mastitis: systematic review and meta-analysis. Prev Vet Med. 2021;188. 10.1016/j.prevetmed.2021.105261. 10.1016/j.prevetmed.2021.105261 33508662

[11] Jamali H, Paydar M, Radmehr B, Ismail S, Dadrasnia A (2015). Prevalence and antimicrobial resistance of *Staphylococcus aureus* isolated from raw milk and dairy products. Food Control.

[12] Varela-Ortiz DF (2018). Antibiotic susceptibility of *Staphylococcus aureus* isolated from subclinical bovine mastitis cases and in vitro efficacy of bacteriophage. Vet Res Commun.

[13] Sasidharan S, Prema B, Yoga Latha L (2011). Antimicrobial drug resistance of *Staphylococcus aureus* in dairy products. Asian Pac J Trop Biomed.

[14] León-Galván MF, et al. Molecular detection and sensitivity to antibiotics and bacteriocins of pathogens isolated from bovine mastitis in family dairy herds of Central mexico. Biomed Res Int. 2015;2015. 10.1155/2015/615153. 10.1155/2015/615153 PMC4359873 25815326

[15] Dai J (2019). Prevalence and characterization of *Staphylococcus aureus* isolated from pasteurized Milk in China. Front Microbiol.

[16] Saeed SI (2022). Prevalence, antimicrobial resistance, and characterization of *Staphylococcus aureus* isolated from subclinical bovine mastitis in East Coast Malaysia. Animals.

[17] Cui M (2020). Emergence of livestock-associated MRSA ST398 from bulk tank milk, China. J Antimicrob Chemother.

[18] Kamaruzzaman NF (2018). Targeting the bacterial protective Armour; challenges and novel strategies in the treatment of microbial biofilm. Materials (Basel).

[19] Singh R, Sahore S, Kaur P, Rani A, Ray P (2016). “Penetration barrier contributes to bacterial biofilm-associated resistance against only select antibiotics , and exhibits genus- , strain- and antibiotic-specific differences,” no. June.

[20] Hall CW, Mah T (2017). “Molecular mechanisms of biofilm-based antibiotic resistance and tolerance in pathogenic bacteria,” no. August 2016.

[21] Fraunholz M, Sinha B (2012). “Intracellular *Staphylococcus aureus*: live-in and let die,” vol. 2, no. April.

[22] Rollin G (2017). Intracellular survival of *Staphylococcus aureus* in endothelial cells: a matter of growth or persistence. Front Microbiol.

[23] Datta S, Roy A (2021). Antimicrobial peptides as potential therapeutic agents: a review. Int J Pept Res Ther.

[24] Bartlett JG, Gilbert DN, Spellberg B (2018). “Seven ways to preserve the miracle of antibiotics,” vol. 56, no. August.

[25] Ngassam-Tchamba C (2020). In vitro and in vivo assessment of phage therapy against *Staphylococcus aureus* causing bovine mastitis. J Glob Antimicrob Resist.

[26] Sengupta I, Bhattacharya P, Talukdar M, Neogi S, Pal SK, Chakraborty S (2019). Bactericidal effect of graphene oxide and reduced graphene oxide: Influence of shape of bacteria. Colloids Interface Sci Commun.

[27] Gupta A, Mumtaz S, Li CH, Hussain I, Rotello VM (2019). Combatting antibiotic-resistant bacteria using nanomaterials. Chem Soc Rev.

[28] Zhang T, Tremblay P. iScience ll Graphene : an antibacterial agent or a promoter of bacterial Proliferation? Science. 2020;23(12):101787. 10.1016/j.isci.2020.101787. 10.1016/j.isci.2020.101787 PMC7701186 33294795

[29] Manuscript A (2018). Nanoscale Horizons.

[30] Liu X, Duan G, Li W, Zhou Z, Zhou R (2017). Membrane destruction-mediated antibacterial activity of tungsten disulfide (WS2). RSC Adv.

[31] Kamaruzzaman NF, Chong SQY, Edmondson-Brown KM, Ntow-Boahene W, Bardiau M, Good L. Bactericidal and anti-biofilm effects of polyhexamethylene Biguanide in models of intracellular and biofilm of Staphylococcus aureus isolated from bovine mastitis. Front Microbiol. 2017;8. 10.3389/fmicb.2017.01518. 10.3389/fmicb.2017.01518 PMC5554503 28848527

[32] Internalization B, Formation B (2013). “*Staphylococcus epidermidis* in orthopedic device infections : the role of *staphylococcus epidermidis* in orthopedic device infections : the role of bacterial internalization in human osteoblasts and biofilm formation,” no. June.

[33] Aureus S (2007). tissue culture assays used to analyze invasion by *Staphylococcus aureus*.

[34] Edwards AM, Massey RC (2011). Invasion of human cells by a bacterial pathogen. J Vis Exp.

[35] Saeed SI (2021). Antibacterial activity of ikarugamycin against intracellular *Staphylococcus aureus* in bovine mammary epithelial cells in vitro infection model.

[36] Xia M, Xie Y, Yu C, Chen G, Li Y, Zhang T (2019). Graphene-based nanomaterials : the promising active agents for antibiotics- independent antibacterial applications. J Control Release.

[37] Lopez A, Liu J (2020). “Covalent and noncovalent functionalization of graphene oxide with DNA for smart sensing,” vol. 2000123.

[38] Zou X, Zhang L, Wang Z, Luo Y (2016). Mechanisms of the antimicrobial activities of Graphene materials. J Am Chem Soc.

[39] Di Giulio M, Zappacosta R, Lodovico D, Campli D, Siani G, Fontana A (2018). Crossm antimicrobial and antibiofilm efficacy of graphene oxide against chronic wound microorganisms.

[40] Wang M, Fan Z, Han H (2021). “Autophagy in Staphylococcus aureus Infection” vol. 11, no. October.

[41] Ek-vitorin JF, Burt JM (2013). Biochimica et biophysica acta structural basis for the selective permeability of channels made of communicating junction proteins ☆. BBA Biomembr.

[42] Foroozandeh P, Aziz AA. Insight into cellular uptake and intracellular trafficking of nanoparticles. Nanoscale Res Lett. 2018;13. 10.1186/s11671-018-2728-6. 10.1186/s11671-018-2728-6 PMC6202307 30361809

[43] Graphene AD (2013). Enhancing cell nucleus accumulation.

[44] Chen Y, et al. Dynamic interactions and intracellular fate of label-free GO within mammalian cells: role of lateral sheet size. bioRxiv. 2019:1–41. 10.1101/805200. 10.1039/d1na00133g PMC9419297 36132849

[45] Mercer J, Helenius A (2009). Virus entry by macropinocytosis. Nat Cell Biol.

[46] Canton J (2018). “Macropinocytosis : new insights into its underappreciated role in innate immune cell Surveillance,” vol. 9, no. October.

[47] Lammel T, Boisseaux P, Fernández-Cruz ML, Navas JM (2013). Internalization and cytotoxicity of graphene oxide and carboxyl graphene nanoplatelets in the human hepatocellular carcinoma cell line Hep G2. Part Fibre Toxicol.

[48] Lammel T, Navas JM (2020). Graphene nanoplatelets spontaneously translocate into the cytosol and physically interact with cellular organelles in the fish cell line. Aquat Toxicol.

[49] Hui L (2014). Availability of the basal planes of graphene oxide determines whether it is antibacterial. ACS Appl Mater Interfaces.

[50] Chen J, Wang X, Han H (2013). A new function of graphene oxide emerge : inactivating phytopathogenic bacterium Xanthomonas oryzae pv. Oryzae.

[51] Zhao J, Wang Z, White JC, Xing B (2014). Graphene in the aquatic environment : adsorption, dispersion, toxicity and transformation graphene in the aquatic environment : adsorption, dispersion, toxicity and transformation.

[52] Wu L (2013). Aggregation kinetics of graphene oxides in aqueous solutions: experiments, mechanisms, and modeling. Langmuir.

[53] Ruiz ON (2011). “Graphene oxide: a nonspeci fi c enhancer of cellular growth,” no. 10.

[54] Garcia-alegria E, et al. Graphene oxide promotes embryonic stem cell differentiation to haematopoietic lineage. Nat Publ Gr. 2016:1–13. 10.1038/srep25917. 10.1038/srep25917 PMC4873758 27197878

[55] Chang Y (2011). In vitro toxicity evaluation of graphene oxide on A549 cells. Toxicol Lett.

[56] Wu X, Tan S, Xing Y, Pu Q, Wu M, Zhao JX (2017). Graphene oxide as an efficient antimicrobial nanomaterial for eradicating multi-drug resistant bacteria in vitro and in vivo. Colloids Surf B Biointerfaces.

[57] Ramalingam V, Raja S, Sundaramahalingam S, Rajaram R (2019). Chemical fabrication of graphene oxide nanosheets attenuates biofilm formation of human clinical pathogens. Bioorg Chem.

[58] Wang X, Han Q, Yu N, Wang T, Wang C, Yang R (2018). GO-AgCl/ag nanocomposites with enhanced visible light-driven catalytic properties for antibacterial and biofilm-disrupting applications. Colloids Surf B Biointerfaces.

[59] Chen Y (2021). Nanoscale Advances free, thin graphene oxide sheets within mammalian.

[60] Nguyen THD, Lin M, Mustapha A (2015). Toxicity of graphene oxide on intestinal bacteria and Caco-2 cells. J Food Prot.

